# Using voice recognition and machine learning techniques for detecting patient‐reported outcomes from conversational voice in palliative care patients

**DOI:** 10.1111/jjns.12644

**Published:** 2025-01-07

**Authors:** Lei Dong, Hideyuki Hirayama, XueJiao Zheng, Kento Masukawa, Mitsunori Miyashita

**Affiliations:** ^1^ Department of Palliative Nursing Health Sciences, Tohoku University Graduate School of Medicine Sendai Miyagi Japan

**Keywords:** machine learning, palliative care, patient‐reported outcomes, symptom assessment, voice recognition

## Abstract

**Aim:**

Patient‐reported outcome measures (PROMs) are increasingly used in palliative care to evaluate patients' symptoms and conditions. Healthcare providers often collect PROMs through conversations. However, the manual entry of these data into electronic medical records can be burdensome for healthcare providers. Voice recognition technology has been explored as a potential solution for alleviating this burden. However, research on voice recognition technology for palliative care is lacking. This study aimed to verify the use of voice recognition and machine learning to automatically evaluate PROMs using clinical conversation voice data.

**Methods:**

We recruited 100 home‐based palliative care patients from February to May 2023, conducted interviews using the Integrated Palliative Care Outcome Scale (IPOS), and transcribed their voice data using an existing voice recognition tool. We calculated the recognition rate and developed a machine learning model for symptom detection. Model performance was primarily evaluated using the F1 score, harmonic mean of the model's positive predictive value, and recall.

**Results:**

The mean age of the patients was 80.6 years (SD, 10.8 years), and 34.0% were men. Thirteen patients had cancer, and 87 did not. The patient voice recognition rate of 55.6% (SD, 12.1%) was significantly lower than the overall recognition rate of 76.1% (SD, 6.4%). The F1 scores for the five total symptoms ranged from 0.31 to 0.46.

**Conclusion:**

Although further improvements are necessary to enhance our model's performance, this study provides valuable insights into voice recognition and machine learning in clinical settings. We expect our findings will reduce the burden of recording PROMs on healthcare providers, increasing the wider use of PROMs.

## 1 INTRODUCTION

Patient‐reported outcome measures (PROMs) are used to assess patients' symptoms and provide a comprehensive view of their health (Calvert et al., 2019; Gilbert et al., 2015). Especially in palliative care, patient‐reported outcomes (PROs) are the primary measures in 61% of intervention trials and 42% of observational studies (Vinches et al., 2020). Patients in palliative care often experience pain symptoms, including physical, psychiatric, social, and spiritual issues (Addington‐Hall et al., 1992). To provide appropriate treatment and care, it is essential to comprehensively evaluate patients' conditions and communicate effectively with them. PROMs are crucial for evaluating symptom management and the health‐related quality of life. PROMs positively impact the quality of life of palliative care patients by improving total pain symptoms and communication between healthcare providers (Etkind et al., 2015; Graupner et al., 2021).

However, integrating PRO data into clinical practice presents several challenges. Patients may encounter difficulties in completing PROMs. Healthcare providers lack the time and knowledge to meaningfully interpret PRO data in clinical practice and cannot act on PRO data. Moreover, there is currently insufficient information technology infrastructure to easily collect PRO data (Nguyen et al., 2021). Furthermore, manual entry of PROMs information into electronic record systems can be burdensome for healthcare providers (Hirayama et al., 2022). The burden of creating nursing records has a weak to moderate correlation with healthcare provider burnout syndrome. In addition, the poor usability of electronic record systems has been linked to documentation burden and burnout syndrome (Gesner et al., 2022). Therefore, it is imperative to address these challenges to facilitate effective integration of PRO data into clinical practice and enhance patient outcomes.

A possible solution to reduce the burden on healthcare providers is to implement speech recognition and machine learning technologies. Voice recognition is primarily used to aid in creating medical records such as endoscopic reports and nurse documentation (Blackley et al., 2019; Hou et al., 2022; Mayer et al., 2021; Takayama et al., 2023). Researchers have developed models combining voice recognition with machine learning to detect symptoms and diagnose diseases from voice data (Chi et al., 2022; De Boer et al., 2023; Horigome et al., 2022; Jothilakshmi, 2014; Kim et al., 2021; Suparatpinyo & Soonthornphisaj, 2023). For instance, some studies have reported the development of machine learning models for automatically classifying the urgency of outpatients (Kim et al., 2021). Additionally, machine learning models have been developed to detect voice pathology, depressive symptoms, autism in children, schizophrenia‐spectrum disorders, and neurocognitive disorders (Chi et al., 2022; De Boer et al., 2023; Horigome et al., 2022; Jothilakshmi, 2014; Suparatpinyo & Soonthornphisaj, 2023).

However, voice recognition technology has not been used to gather PROs for palliative care. Therefore, to evaluate the potential of speech recognition technology in palliative care clinical settings, we assessed existing voice recognition tools and developed a machine model by analyzing speech data from conversations between healthcare providers and palliative care patients regarding total pain to detect symptoms.

## 2 METHODS

### 2.1 Study design

This cross‐sectional study was approved by the Tohoku University Graduate School of Medicine Institutional Review Board (approval number: 2022‐1‐888).

### 2.2 Study population

Between February 1 and May 31, 2023, we recruited patients receiving palliative care at the Houen Home Care Clinic, which provides regular home‐visit treatment from medical professionals. The following criteria were applied for eligibility: (1) receiving palliative care at home, (2) aged 18 years or older, and (3) able to speak Japanese and understand the written Japanese instructions. (4) Patients with dementia were included in the study if their primary care physicians and researchers deemed them capable of effective communication, active participation, and adequately understanding and completing the PROMs. Exclusion criteria included: (1) apparent consciousness disorders; (2) severe physical symptoms such as pain, respiratory distress, fatigue, nausea, or vomiting, which were deemed unsuitable for participation by the attending physician or researcher; and (3) severe psychiatric symptoms, which were deemed unsuitable for participation by the attending physician or researcher.

We recruited 100 patients for this study. Previous studies that estimated emotions from speech data used the conversational speech of 96 people (Shimura et al., 2010). The database used to assess emotions from speech data consists of 300–5000 utterances (Akçay & Oğuz, 2020). This study used a 23‐item patient‐reported outcome measure, with each response equivalent to one utterance. Therefore, it was possible to obtain more than 23 utterances per patient if we assumed that each response was an utterance. Our analysis found that the 100 patients included in the study could generate 2300 utterances, meeting the criteria for the sample size.

### 2.3 Measurements

#### 2.3.1 Patient reported outcomes

This study used the 3‐day version of the Integrated Palliative Care Outcome Scale (IPOS) (Tables S1 and S2) as the patient‐reported outcome measure. A Japanese version of the IPOS was developed and validated for reliability and validity in cancer and noncancer patients (Ishii et al., 2023; Sakurai et al., 2019; Schildmann et al., 2016). The IPOS assesses physical, emotional, and communication/practical symptoms. Each item consists of a five‐point level from 0 to 4, with the user selecting the closest match based on the description provided for each level. For example, in the case of pain, 0 was defined as “not at all,” 1 as “slight,” 2 as “moderate,” 3 as “severe,” and 4 as “overwhelming.”

#### 2.3.2 Patients' characteristics

We collected the patients' basic information from the electronic medical record system, which included their gender, age, primary illness, previous medical history, cognitive function level, and required level of care and support.

### 2.4 Collection of voice data and transcription

We conducted interviews in patients' homes and nursing homes. Previous studies have utilized iPad devices for their efficacy in home health care as recording devices (Crichton et al., 2012; Riley, 2013; Taylor et al., 2015). Therefore, we used a tablet (iPad Air 2, Apple, USA) as our recording device. Before recording, we explained the interview contents to the patients and obtained their consent. The interview, including all responses to the IPOS questions between patients and healthcare providers, was recorded from start to finish. We recruited two native Japanese speakers from Tohoku University to manually transcribe recorded voice data to generate human‐transcribed scripts.

We also used a voice recognition tool, “Ami Voice Medical Conference” (Advanced Media, Japan), to transcribe the voice data into text automatically. This tool was designed to create medical meeting records. Users can choose between medical and general purpose dictionaries for voice recognition. The medical dictionary contains the names of diseases, symptoms, and drugs. The system operates independently to ensure security. Its successful use in voice recognition research (Kinoshita, 2021; Shikino et al., 2023) makes it a valuable choice for future applications.

### 2.5 Statistical analysis

#### 2.5.1 Calculating voice recognition rate

We compared the automatically transcribed text data with the correct text data and counted the error characters to determine the voice recognition rate. Voice recognition rate is the proportion of words correctly recognized by the tool to the total number of words spoken. A higher voice recognition rate indicates better model performance. Voice recognition rate is defined as follows:

$$\text{Voice Recognition Rate}=\frac{N-D-S-I}{N},$$

where *N* is the total number of words in the correct text data, and *D*, *S*, and *I* are the deletion, substitution, and insertion error characters in the automatically transcribed text data.

#### 2.5.2 Model development and performance evaluation

We preprocessed the automatically transcribed text data before inputting them into the machine learning model. Labels were assigned to each item based on the IPOS questionnaire scores. The scoring criteria were as follows: scores less than 2 were considered negative, and scores of 2 or higher were considered positive. A score of 2 or higher on the IPOS was used as the cutoff value, indicating moderate or severe symptoms that may require medication adjustment, treatment, or assistance from healthcare providers (Sakurai et al., 2019).

We used Vertex AI (Google LLC, USA), an auto‐machine learning tool for text classification, to develop a machine learning model that detects total pain symptoms by performing binary classification with a single label.

First, the labeled data were uploaded to a Google Cloud Storage bucket. Second, the text data from the automatic transcription were split into datasets for training (40%), validation (20%), and testing (40%). After splitting the dataset, model training was initiated using the automatically transcribed text data. In the auto‐machine learning training process, various tasks, such as preprocessing, machine learning method selection, and hyperparameter optimization, were performed automatically. The best model was selected based on its performance in the training and validation data (Opara et al., 2022).

In reference to previous studies (Kim et al., 2021), we used the F1 score as the primary endpoint to evaluate model performance. The F1 score measures the accuracy and overall performance of the model. This is the harmonic mean of the model's positive predictive value (precision) and recall (Chicco & Jurman, 2020). The F1 score close to 1.0 indicates that both precision and recall are high, providing a balance between the two metrics. The F1 score is defined as follows:

$$F1=2\times \frac{\text{Precision}\times \text{Recall}}{\;\left(\text{Precision}+\text{Recall}\right)}.$$

Furthermore, we calculated the sensitivity (recall) and positive predictive value (precision). Sensitivity (recall) measures how well a model identifies all the relevant cases in a dataset. The positive predictive value (precision) measures the accuracy of the optimistic predictions made by the model. This represents the proportion of correct identifications. The sensitivity of the model was adjusted to approximately 80% for performance evaluation. The training and evaluation processes were repeated thrice, and the average values of each evaluation metric were calculated.

Finally, we analyzed the correlation between patient disease and the voice recognition rate. Statistical analysis was conducted using JMP® Pro 17, and we performed the Kruskal–Wallis test with a significance level of less than 5%.

## 3 RESULTS

### 3.1 Patients' characteristics

Table 1 shows the patient characteristics of the total sample. A total of 100 patients were enrolled and analyzed. The mean age was 80.6 years (standard deviation [SD] 10.8 years); 34 patients were men, 13 had cancer, and 87 had no cancer. Twenty‐three patients were diagnosed with dementia. The cognitive function score of these patients ranged from normal to rank 2b (Tago et al., 2021). Ninety‐four patients needed care. The mean interview duration was 12 min 23 s (SD, 6 min 14 s).

**TABLE 1:** Patient's characteristic.

Total sample (*N* = 100)			
	*n* (%)		*n* (%)
Age (years) (mean ± SD)	(80.5 ± 10.8)	Cancer diseases (*n* = 13)	
Gender		Head and neck	1 (7.7)
Men	34 (34.0)	Breast	1 (7.7)
Women	66 (66.0)	Lung	1 (7.7)
Primary illness		Gastric and esophageal	2 (15.4)
Cancer diseases	13 (13.0)	Hepatobiliary and pancreatic	1 (7.7)
Non‐cancer disease	87 (87.0)	Colon and rectum	3 (23.1)
The level of care and support needed		Urology	2 (15.4)
Support level 1	3 (3.0)	Uterus and ovary	2 (15.4)
Support level 2	3 (3.0)	Non‐cancer disease (*n* = 87)	
Care level 1	35 (35.0)	Dementia	23 (26.4)
Care level 2	22 (22.0)	Cardiovascular disease	15 (17.2)
Care level 3	11 (11.0)	Neurological diseases	11 (12.6)
Care level 4	18 (18.0)	Hypertension	6 (7.2)
Care level 5	8 (8.0)	Diabetes	5 (6.1)
Location of interview		Stroke	5 (6.1)
Nursing home	67 (67.0)	Others	22 (25.3)
Private residence	33 (33.0)		
Interview duration (mean ± SD)		(12 min 23 s ± 6 min 14 s)	

Abbreviation: SD, standard deviation.

In addition to the characteristics above, the data collection process was subjected to detailed analysis. Of the 100 interviews conducted, 33 were carried out in private residences and 67 in nursing homes. The interviews were conducted in the patients' living spaces, such as their bedrooms or common areas within nursing facilities, to ensure their comfort and accessibility. The mean number of individuals present during each interview was three: two nurses conducted the interview session with the patient. In certain cases, supplementary individuals, such as caregivers, family members, or rehabilitation specialists, were also present. The tablet (iPad Air2) used for recording was on a table near patients' chairs, on the floor near the feet of patients in wheelchairs, or on the bedside table near the heads of bedridden patients.

### 3.2 Distribution of IPOS items

The responses to the IPOS questionnaire are presented in Table 2. The highest number of responses to all the questions was zero (not at all). The number of patients with an IPOS score of 2 (moderate) or more for each symptom was as follows: 23 for the physical symptom “pain,” 26 for the psychological symptom “anxiety,” 21 for “depression,” 20 for the spiritual pain “feeling at peace,” and 22 for the social distress “practical matters.”

**TABLE 2:** Distribution of IPOS items.

			*N* = 100		
Subscale/Items	Not at all (0)	Slight (1)	Moderate (2)	Severe (3)	Overwhelming (4)
Physical symptom					
Pain	54	23	14	3	6
Psychological symptoms					
Patient anxiety	61	13	11	2	13
Depression	61	18	13	2	6
Spiritual pain					
Feeling at peace	55	25	9	6	5
Social pain					
Practical matters	50	28	16	5	1

Abbreviation: IPOS, Integrated Palliative Care Outcome Scale.

### 3.3 Speech recognition rate

**FIGURE 1:**
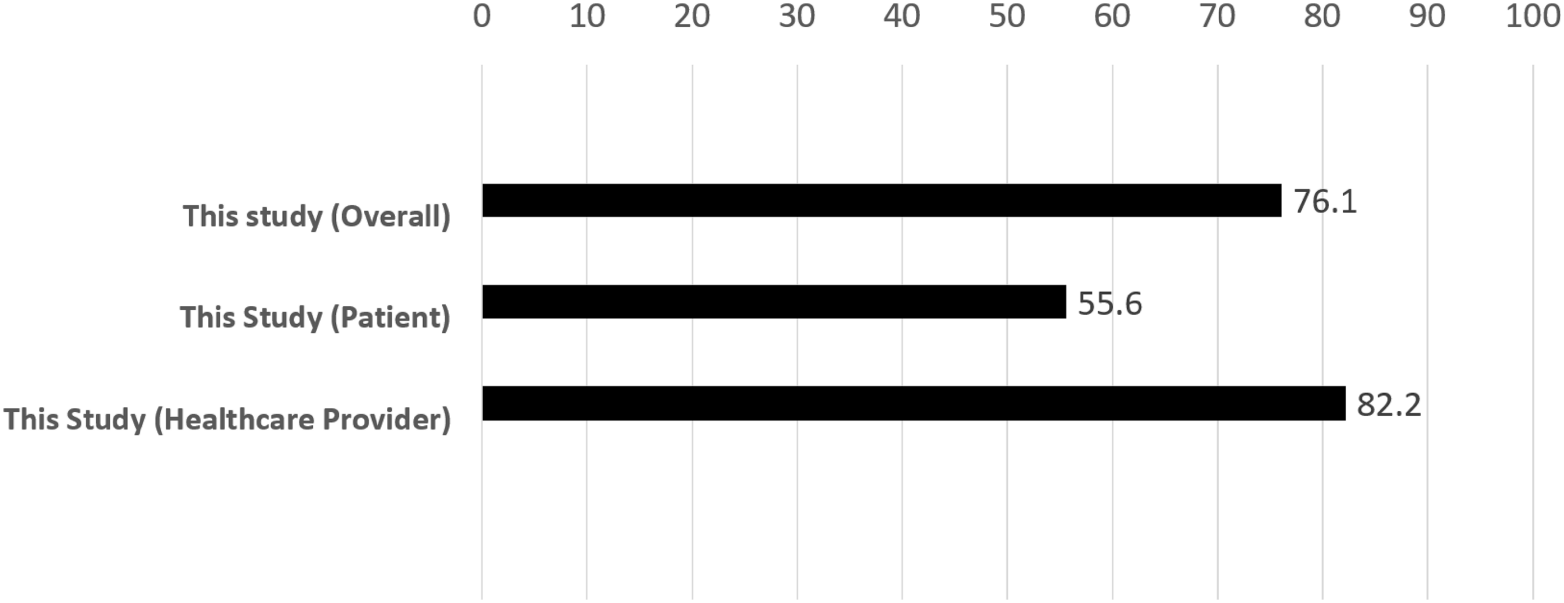
Voice recognition rate (%).

**FIGURE 2:**
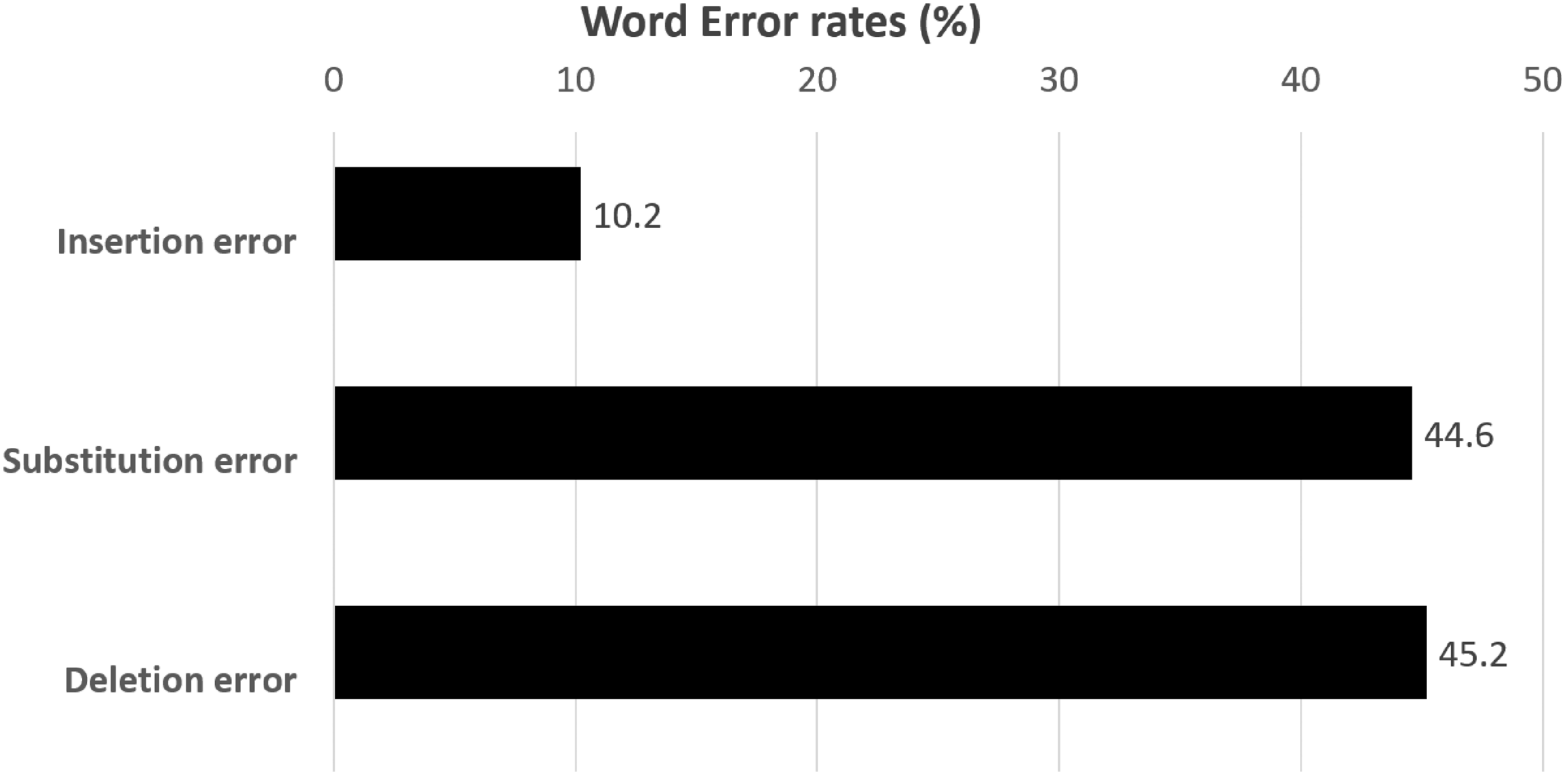
Breakdown of word error rate (%).

Figure 1 shows the speech recognition rate results. The overall recognition rate was 76.1% (SD, 6.4%), with 55.6% (SD, 12.1%) for patients and 82.2% (SD, 6.2%) for healthcare providers. Word error rates were categorized as deletion errors (45.2%), substitution errors (44.6%), and insertion errors (10.2%) (Figure 2). Table 3 shows the speech recognition rates of the patients, which varied according to their disease. Patients who had suffered strokes had the lowest recognition rate at 48.3% (SD, 22.1%).

**TABLE 3:** Voice recognition rate of patients with different diseases.

Diseases	*n*	Voice recognition rate(%) (mean ± SD)	*p*
Cancer	13	50.8 ± 11.5	.515
Dementia	23	56.9 ± 10.7	
Cardiovascular disease	15	58.1 ± 10.1	
Neurological diseases	11	53.6 ± 9.4	
Hypertension	6	59.1 ± 13.1	
Diabetes	5	53.7 ± 15.4	
Stroke	5	48.3 ± 22.1	
Others	22	58.5 ± 10.9	

Abbreviation: SD, standard deviation.

### 3.4 Model performance

Table 4 presents the results of the performance evaluation of the symptom detection model using the automatically transcribed text data from the voice recognition tool. The physical symptom “pain” achieved an F1 score of 0.36, sensitivity of 0.74, and positive predictive accuracy of 0.24. The psychological symptom “anxiety” achieved an F1 score of 0.46, sensitivity of 0.39, and positive predictive value of 0.59. Depression achieved an F1 score of 0.45, sensitivity of 0.75, and positive predictive value of 0.32. The spiritual “feeling at peace” achieved an F1 score of 0.43, sensitivity of 0.67, and positive predictive value of 0.33. The social distress “practical matters” achieved an F1 score of 0.31, sensitivity of 0.45, and positive predictive value of 0.34.

**TABLE 4:** Model performance.

Evaluation metrics	F1 score	Sensitivity (recall)	Specificity	Positive predictive value (precision)	Negative predictive value	Average precision
Pain	0.36	0.74	0.32	0.24	0.82	0.41
Patient anxiety	0.46	0.39	0.90	0.59	0.81	0.59
Depression	0.45	0.75	0.58	0.32	0.90	0.47
Feeling at peace	0.43	0.67	0.65	0.33	0.89	0.49
Practical matters	0.31	0.45	0.66	0.34	0.81	0.37

## 4 DISCUSSION

This is the first study to evaluate the voice recognition rate and develop a machine learning model for detecting five total pain symptoms using patient–healthcare provider conversational speech data on PROMs obtained in a palliative care clinical setting. This study has two key findings. First, the voice recognition rate of existing voice recognition tools was approximately 75%, indicating that there is still room for improvement in home‐visit settings. Second, the performance of the machine learning model was inadequate, with an F1 score of less than 0.5 for all symptom items.

The voice recognition rate in this study (55%–82%) was lower than that in a previous study compared with the Kaldi and Google Cloud Speech API, two widely used voice recognition tools, using the Corpus of Spontaneous Japanese (81%–90%) (Kimura et al., 2019). There are three possible reasons for this discrepancy: First, the interview environment was the clinical setting of the home visit, which affected the quality of the recorded speech. Proper positioning of the interviewee is a critical factor in reducing the error rate of speech recognition systems (Wölfel et al., 2005), and background noise in the medical environment can reduce the voice recognition rate (Alapetite, 2008). This study was conducted in the clinical setting of home visits, mainly in patients' private residences and nursing homes. The interview setting typically consisted of two nurses in the interview team and the patient, comprising three people in the room. However, depending on the patient's needs and circumstances, additional individuals such as caregivers, family members, or rehabilitation specialists were occasionally present, which may have influenced the dynamics of the interviews. The recording process was limited by several factors related to the positioning of the tablet and the physical environment. Tablet positioning varied depending on the patient's condition: The tablet was placed on a nearby table for patients seated in chairs. The tablet was positioned on the floor near patients in wheelchairs' feet and on the bedside near their heads for bedridden patients. These setups were chosen to ensure the best possible audio capture within the constraints of the environment. However, the positioning of the equipment, the posture and distance during conversations with the patients, and the unavoidable background noise in home‐visit settings may have negatively affected the speech recognition rate. Second, most participants in this study were in their 80s and voice changes related to aging were found to impact communication negatively (Lindstrom et al., 2023). Voice data from older adults may have a lower speech recognition rate than that from younger individuals (Werner et al., 2019). The characteristics of speech in older people include age‐related loss of muscle strength in articulatory organs, resulting in reduced speech intensity during speech acts and slower lip and tongue movements, leading to unclear speech (Vipperla et al., 2008). Furthermore, in this study, the cognitive function levels of the 23 patients diagnosed with dementia range from normal to 2b, defined as a state where symptoms, behaviors, or communication difficulties that interfere with daily life are somewhat present at home (Tago et al., 2021). Given the complexity of the symptoms associated with dementia, it seems probable that these factors introduced bias into the interview conversation content and recognition results. Several chronic conditions, including dementia, stroke, and Parkinson's disease, are frequently associated with dysphonia, which potentially impairs speech quality (Kost & Sataloff, 2020). Our analysis also confirmed that the voice recognition rate among stroke patients was the lowest among patients with other diseases, although the *p*‐value was greater than 0.05. Third, the voice recognition tool may have affected the results; this tool was designed for medical conferences and may not have been as effective for interviews in home‐visit settings. This discrepancy may have contributed to the lower recognition rates. As home‐visiting care frequently involves older adults, it is essential to consider their speech characteristics and develop a speech recognition tool trained explicitly on the acoustic characteristics of older adults. In addition, constraints related to posture, recording equipment location, background noise, and distance in the clinical setting must be improved to enhance speech data quality in home‐visit medical environments. Moreover, techniques for managing environmental noise in clinical environments, such as spectral subtraction (Kleinschmidt et al., 2011), noise reduction (Garg & Jain, 2016), and source separation (Liu et al., 2023), should be considered. These techniques can potentially improve the voice recognition rates.

The performance of the model for all symptom detections had an F1 score of less than 0.5. This was deemed insufficient compared with the model performance (F1 score = 0.72) of a similar previous study (Kim et al., 2021). The poor performance of the model can be attributed to two factors. First, it is imperative to enhance the quality of input data. It should be noted that the model was developed using text data transcribed by a voice recognition tool. The overall voice recognition rate was not optimal, particularly for patient speech (55%). The use of low‐quality input data transcribed using existing voice recognition tools presents a significant challenge. Previous research has demonstrated that the overall performance of machine learning models declines as the character error rate increases (Kim et al., 2021). The model's performance may have been affected by the use of text data with low recognition rates. Therefore, improving the quality of the input data is expected to enhance the model's performance to a certain extent. Second, it is crucial to consider the impact of imbalanced datasets. Imbalanced data refer to a skewed data structure with a deficient number of negative or positive data on one side. After analyzing the distribution of symptom scores in the IPOS, we observed significant differences in the number of samples labeled as negative and positive for each item. In machine learning, an imbalanced dataset can cause a model to focus on the majority class and ignore the minority class. This can lead to overlearning of the majority of class features and poor performance on the test data (Liu et al., 2023). To address the issue of imbalanced datasets, various methods, such as undersampling and oversampling (Mohammed et al., 2020), can be employed to balance the data set. Furthermore, auto‐machine learning makes it difficult to adjust the model directly and set the parameters; therefore, developing a model using alternative methods may lead to higher performance.

Our study has several limitations. First, voice data are scarce and insufficient for training machine learning models. A small sample size may limit the model training and increase the risk of overtraining. Second, this study was based on auto‐machine learning (black box model); therefore, it is not easy to adjust the model details, and the interpretability of the model is limited. This limitation may affect its application in clinical practice and healthcare providers' decision‐making. Therefore, future work should include methods to improve interpretability, such as integrating auto‐machine learning models with SHAP (SHapley Additive exPlanations) (Sun et al., 2023) or using other non‐auto‐machine learning approaches to improve interpretability. To achieve this objective, close collaboration with healthcare providers is essential. Third, the only recording device used in this study was an iPad. Future studies should consider using alternative devices, such as a headset microphone, to improve audio quality. Lastly, this study was conducted in a home‐visit setting and may not be generalizable to other palliative care settings (e.g., palliative care units, clinics, and wards). Therefore, it is necessary to investigate and evaluate voice recognition techniques in other palliative care settings.

## 5 CONCLUSIONS

Existing voice recognition tools still have poor voice recognition rates for speech data when listening to patients' symptoms by healthcare providers in home‐visit settings. However, this study provides the latest practical insights into voice recognition in a palliative care clinical setting. Although our machine learning model still requires further improvements to be applied in clinical practice, we expect it to reduce the burden of recording PROMs on healthcare providers and increase the use of PROMs more widely. This study enhances the usefulness of machine learning and voice recognition technologies in palliative care.

## AUTHOR CONTRIBUTIONS

All the authors contributed to the study design. Lei Dong contributed to collecting and assembling the data and writing the manuscript, Kento Masukawa and Hideyuki Hirayama contributed to the statistical analysis, and Mitsunori Miyashita contributed to revising the manuscript. All authors discussed the results and contributed to the final manuscript.

## FUNDING INFORMATION

This study was supported by the JSPS KAKENHI (grant number 22K11240). The funder had no role in the study design, data collection, analysis and interpretation, writing of the report, or decision to submit the article for publication. The study was conducted in accordance with the principles of the Declaration of Helsinki and the ethical guidelines for medical research involving human subjects. Written informed consent was obtained from all the patients.

## CONFLICT OF INTEREST STATEMENT

The authors declare no conflicts of interest.

## Supporting information


**Data S1.** Supporting information.

**Data S2.** Supporting information.
