# Supplementary material for: Using voice recognition and machine learning techniques for detecting patient‐reported outcomes from conversational voice in palliative care patients
Source: Jpn J Nurs Sci. 2025 Jan 7;22(1):e12644. doi: 10.1111/jjns.12644 (PMC11707305; doi:10.1111/jjns.12644)
Supplement: Supplementary file 1 — Data S1. Supporting information. [file JJNS-22-e12644-s001.docx]

**Supplementary Table 1**

**Integrated Palliative care Outcomes Scale Patient Version**

Patient name : …………………………………………………

Date (dd/mm/yyyy) : …………………………………………………

*Patient number* : ………………………………… *(for staff use)*

Q1. What have been your main problems or concerns over the past 3 days?

1. ............................................................................................................................................

2. ............................................................................................................................................

3. ............................................................................................................................................

Q2. Below is a list of symptoms, which you may or may not have experienced. For each symptom, please tick one box that best describes how it has affected you over the past 3 days.

|  | Not at all | Slightly | Moderately | Severely | Over-whelmingly |
| --- | --- | --- | --- | --- | --- |
| Pain | 0 | 1 | 2 | 3 | 4 |
| Shortness of breath | 0 | 1 | 2 | 3 | 4 |
| Weakness or lack of energy | 0 | 1 | 2 | 3 | 4 |
| Nausea (feeling like you are going to be sick) | 0 | 1 | 2 | 3 | 4 |
| Vomiting (being sick) | 0 | 1 | 2 | 3 | 4 |
| Poor appetite | 0 | 1 | 2 | 3 | 4 |
| Constipation | 0 | 1 | 2 | 3 | 4 |
| Sore or dry mouth | 0 | 1 | 2 | 3 | 4 |
| Drowsiness | 0 | 1 | 2 | 3 | 4 |
| Poor mobility | 0 | 1 | 2 | 3 | 4 |
| Please list any other symptoms not mentioned above, and tick one box to show how they have affected you over the past 3 days. | | | | | |
| 1. | 0 | 1 | 2 | 3 | 4 |
| 2. | 0 | 1 | 2 | 3 | 4 |
| 3. | 0 | 1 | 2 | 3 | 4 |

Over the past 3 days:

|  | *Not at all* | *Occasionally* | *Sometimes* | *Most of the time* | *Always* |
| --- | --- | --- | --- | --- | --- |
| Q3. Have you been feeling anxious or worried about your illness or treatment? | 0 | 1 | 2 | 3 | 4 |
| Q4. Have any of your family or friends been anxious or worried about you? | 0 | 1 | 2 | 3 | 4 |
| Q5. Have you been feeling depressed? | 0 | 1 | 2 | 3 | 4 |
|  | *Always* | *Most of the time* | *Sometimes* | *Occasionally* | *Not at all* |
| Q6. Have you felt at peace? | 0 | 1 | 2 | 3 | 4 |
| Q7. Have you been able to share how you are feeling with your family or friends as much as you wanted? | 0 | 1 | 2 | 3 | 4 |
| Q8. Have you had as much information as you wanted? | 0 | 1 | 2 | 3 | 4 |
|  | *Problems addressed/ No problems* | *Problems mostly addressed* | *Problems partly addressed* | *Problems hardly addressed* | *Problems not addressed* |
| Q9. Have any practical problems resulting from your illness been addressed? (such as financial or personal) | 0 | 1 | 2 | 3 | 4 |
|  | *On my own* | *With help from a friend or relative* | | | *With help from a member of staff* |
| Q10. How did you complete this questionnaire? |  |  | | |  |

*If you are worried about any of the issues raised on this questionnaire
then please speak to your doctor or nurse.*
