# Supplementary material for: Using voice recognition and machine learning techniques for detecting patient‐reported outcomes from conversational voice in palliative care patients
Source: Jpn J Nurs Sci. 2025 Jan 7;22(1):e12644. doi: 10.1111/jjns.12644 (PMC11707305; doi:10.1111/jjns.12644)
Supplement: Supplementary file 2 — Data S2. Supporting information. [file JJNS-22-e12644-s002.docx]

**Supplementary Table 2**

時間：

場所：

実施者：

研究ＩＤ：

**Integrated Palliative care Outcomes Scale Patient Japanese Version**

IPOSアンケート用紙

　　　　　　　　　　　　　　　　　　　　　　　　　　　　2022年12月9日　作成

Q1. この3日間、主に大変だったことや気がかりは何でしたか？

1. ............................................................................................................

2. ............................................................................................................

3. ............................................................................................................

Q2.以下はあなたが経験したかもしれない症状のリストです。それぞれの症状について、この3日間、どれくらい生活に支障があったか最もよく表しているものに一つだけチェックしてください。

|  | 全く支障は  なかった | 少しあった（気にならなかった） | 中くらい  あった  （いくらか支障がでた） | とても  あった  （大きな支障がでた） | 耐えられないくらいあった  （他のことを考えられなかった） |
| --- | --- | --- | --- | --- | --- |
| 痛み | 0 | 1 | 2 | 3 | 4 |
| 息切れ（息苦しさ） | 0 | 1 | 2 | 3 | 4 |
| 力や元気が出ない感じ（だるさ） | 0 | 1 | 2 | 3 | 4 |
| 吐き気　(吐きそうだった) | 0 | 1 | 2 | 3 | 4 |
| 嘔吐　(実際に吐いた) | 0 | 1 | 2 | 3 | 4 |
| 食欲不振 | 0  （通常の食欲） | 1 | 2 | 3 | 4  （食欲が全くない） |
| 便秘 | 0 | 1 | 2 | 3 | 4 |
| 口の痛みや渇き | 0 | 1 | 2 | 3 | 4 |
| 眠気 | 0 | 1 | 2 | 3 | 4 |
| 動きにくさ | 0 | 1 | 2 | 3 | 4 |
| 上記以外の症状があれば記入し、この3日間、どれくらい生活に支障があったか一つだけチェックしてください。 | | | | | |
| 1.＿＿＿＿＿＿＿＿＿＿＿＿＿ | 0 | 1 | 2 | 3 | 4 |
| 2.＿＿＿＿＿＿＿＿＿＿＿＿＿ | 0 | 1 | 2 | 3 | 4 |
| 3.＿＿＿＿＿＿＿＿＿＿＿＿＿ | 0 | 1 | 2 | 3 | 4 |

この3日間についてお聞きします

|  | *全くなし* | *たまに* | *ときどき* | *たいてい* | *いつも* |
| --- | --- | --- | --- | --- | --- |
| Q3. 病気や治療のことで不安や心配を感じていましたか？ | 0 | 1 | 2 | 3 | 4 |
| Q4.家族や友人は、あなたのことで不安や心配を感じていた様子でしたか？ | 0 | 1 | 2 | 3 | 4 |
| Q5.気分が落ち込むことはありましたか？ | 0 | 1 | 2 | 3 | 4 |
|  | *いつも* | *たいてい* | *ときどき* | *たまに* | *全くなし* |
| Q6.気持ちは穏やかでいられましたか？ | 0 | 1 | 2 | 3 | 4 |
| Q7.あなたの気持ちを家族や友人に十分に分かってもらえましたか？ | 0 | 1 | 2 | 3 | 4 |
| Q8.治療や病気について、十分に説明がされましたか？ | 0 | 1 | 2 | 3 | 4 |
|  | 全て対応されている／  問題がない | 大部分対応  されている | 一部対応されている | ほとんど対応されていない | 全く対応さ  れていない |
| Q9. 病気のために生じた、気がかりなことに対応してもらえましたか？  （経済的なことや個人的なことなど） | 0 | 1 | 2 | 3 | 4 |

|  | 自分で | 友人や家族に手伝って  もらって | スタッフに手伝ってもらって |
| --- | --- | --- | --- |
| Q10.どのようにしてこの質問票に答えましたか？ |  |  |  |
